# *Butyricimonas* is a key gut microbiome component for predicting postoperative recurrence of esophageal cancer

**DOI:** 10.1007/s00262-023-03608-y

**Published:** 2024-01-27

**Authors:** Koji Otsuka, Junya Isobe, Yoshiyuki Asai, Tomohisa Nakano, Kouya Hattori, Tomotake Ariyoshi, Takeshi Yamashita, Kentaro Motegi, Akira Saito, Masahiro Kohmoto, Masahiro Hosonuma, Atsuo Kuramasu, Yuta Baba, Masakazu Murayama, Yoichiro Narikawa, Hitoshi Toyoda, Eiji Funayama, Kohei Tajima, Midori Shida, Yuya Hirasawa, Toshiaki Tsurui, Hirotsugu Ariizumi, Tomoyuki Ishiguro, Risako Suzuki, Ryotaro Ohkuma, Yutaro Kubota, Takehiko Sambe, Mayumi Tsuji, Satoshi Wada, Yuji Kiuchi, Shinichi Kobayashi, Atsushi Horiike, Satoru Goto, Masahiko Murakami, Yun-Gi Kim, Takuya Tsunoda, Kiyoshi Yoshimura

**Affiliations:** 1 Showa University Hospital Esophageal Cancer Center, Esophageal Surgery, Tokyo, Japan; 2 Department of Hospital Pharmaceutics, School of Pharmacy, Showa University, Tokyo, Japan; 3 Department of Systems Bioinformatics, Graduate School of Medicine, Yamaguchi University, Yamaguchi, Japan; 4 AI Systems Medicine Research and Training Center, Graduate School of Medicine, Yamaguchi University and Yamaguchi University Hospital, Yamaguchi, Japan; 5 Research Center for Drug Discovery and Faculty of Pharmacy and Graduate School of Pharmaceutical Sciences, Keio University, Tokyo, Japan; 6 Division of Biochemistry, Faculty of Pharmacy and Graduate School of Pharmaceutical Sciences, Keio University, Tokyo, Japan; 7 Division of Medical Oncology, Department of Medicine, Showa University School of Medicine, Tokyo, Japan; 8 Department of Clinical Immuno Oncology, Clinical Research Institute for Clinical Pharmacology and Therapeutics, Showa University, Tokyo, Japan; 9 Department of Pharmacology, Showa University School of Medicine, Tokyo, Japan; 10 Pharmacological Research Center, Showa University, Tokyo, Japan; 11 Department of Otorhinolaryngology-Head and Neck Surgery, Showa University School of Medicine, Tokyo, Japan; 12 Department of Orthopedic Surgery, School of Medicine, Showa University, Tokyo, Japan; 13 Division of Pharmacology, Department of Pharmacology, School of Pharmacy, Showa University, Tokyo, Japan; 14 Department of Gastroenterological Surgery, Tokai University School of Medicine, Tokyo, Japan; 15 Division of Clinical Pharmacology, Department of Pharmacology, Showa University School of Medicine, Tokyo, Japan; 16 Department of Clinical Diagnostic Oncology, Clinical Research Institute for Clinical Pharmacology and Therapeutics, Showa University, Tokyo, Japan; 17 Clinical Research Institute for Clinical Pharmacology and Therapeutics, Showa University, Tokyo, Japan

**Keywords:** *Actinomyces*, *Butyricimonas*, Clinical efficacy, Esophageal cancer, Gut microbiome, Machine learning analysis

## Abstract

**Background:**

Recently, intestinal bacteria have attracted attention as factors affecting the prognosis of patients with cancer. However, the intestinal microbiome is composed of several hundred types of bacteria, necessitating the development of an analytical method that can allow the use of this information as a highly accurate biomarker. In this study, we investigated whether the preoperative intestinal bacterial profile in patients with esophageal cancer who underwent surgery after preoperative chemotherapy could be used as a biomarker of postoperative recurrence of esophageal cancer.

**Methods:**

We determined the gut microbiome of the patients using 16S rRNA metagenome sequencing, followed by statistical analysis. Simultaneously, we performed a machine learning analysis using a random forest model with hyperparameter tuning and compared the data obtained.

**Results:**

Statistical and machine learning analyses revealed two common bacterial genera, *Butyricimonas* and *Actinomyces*, which were abundant in cases with recurrent esophageal cancer. *Butyricimonas* primarily produces butyrate, whereas *Actinomyces* are oral bacteria whose function in the gut is unknown.

**Conclusion:**

Our results indicate that *Butyricimonas* spp. may be a biomarker of postoperative recurrence of esophageal cancer. Although the extent of the involvement of these bacteria in immune regulation remains unknown, future research should investigate their presence in other pathological conditions. Such research could potentially lead to a better understanding of the immunological impact of these bacteria on patients with cancer and their application as biomarkers.

**Supplementary Information:**

The online version contains supplementary material available at 10.1007/s00262-023-03608-y.

## Introduction

Esophageal cancer is the sixth most common cause of cancer-related deaths worldwide [1]. Although surgery is one of the most effective treatments for esophageal cancer, it involves a complex procedure that is associated with considerable morbidity and mortality [2–4]. Recent advances in minimally invasive surgery have reduced the incidence of cardiopulmonary complications and pain after esophagectomy. At Showa University Hospital, thoracoscopic surgery, which is considered to be less invasive, is performed for all cases. Establishing a strategy for preventing recurrence, even after such high-quality surgery, is of great interest; however, predicting the recurrence of cancer without burdening the patient is difficult.

Recently, the efficacy of immune checkpoint inhibitors (ICIs) in the treatment of recurrent and unresectable esophageal cancers has been demonstrated [5], highlighting the importance of the immune system in the control of esophageal cancer. As intestinal bacteria significantly affect the body immunity, we wondered whether the intestinal bacterial profile could be used as a predictive marker for postoperative recurrence of esophageal cancer (Table 1).

**Table 1:** Patient background by recurrence and non-recurrence groups

Recurrence		Recurrence	Non-recurrence
Age, mean (years)		51 ≦ Age ≦ 80 (66)	40 ≦ Age ≦ 85 (67)
Sex	Male	16	22
	Female	2	11
pTNM stage	Stage 0, *n*(%)	0 (0)	5 (15.2)
	Stage IA, *n*(%)	2 (11.1)	11 (33.3)
	Stage IB, *n*(%)	1 (5.6)	2 (6.1)
	Stage IIA, *n*(%)	1 (5.6)	2 (6.1)
	Stage IIB, *n*(%)	2 (11.1)	9 (27.3)
	Stage IIIA ,*n*(%)	2 (11.1)	2 (6.1)
	Stage IIIB ,*n*(%)	5 (27.8)	2 (6.1)
	Stage IIIC ,*n*(%)	2 (11.1)	0 (0)
	Stage IV ,*n*(%)	3 (16.7)	0 (0)
Complications		11	30

The human intestinal mucosa is home to more than 40 trillion bacteria [6, 7]. Gut bacteria contribute to the maintenance of host homeostasis and are associated with the development and treatment of various diseases, such as allergic and inflammatory bowel diseases [8–10]. The intestinal microbiome also plays a role in the development and treatment of cancer [11, 12]. Moreover, the gut microbiome influences the efficacy of cancer treatment. For instance, in malignant melanoma and lung cancer, the gut microbiome is a prognostic predictor of the response to ICIs. *Akkermansia muciniphila* and *Bifidobacterium* have been reported to modulate immune responses against cancer and enhance the therapeutic efficacy of ICIs [13, 14]. On the contrary, intestinal bacteria belonging to the *Clostridia* and *Lactobacillus* genera have been implicated in postoperative recurrence of colorectal cancer. However, although some differences in intestinal bacterial profiles have been considered to be favorable or unfavorable for cancer treatment in different reports and regions, there is no consensus in this regard [15]. Nevertheless, based on the abovementioned reports, we hypothesized that intestinal bacteria could serve as a prognostic factor for the postoperative recurrence of esophageal cancer.

If specific intestinal bacteria can be used as predictive biomarkers for recurrence of esophageal cancer after surgery, stool material, which is normally discarded, can serve as a convenient sample for an easy assessment of biomarkers for predicting the prognosis of esophageal cancer. This would also lead to a better understanding of the immunological mechanisms involved in the esophageal cancer microenvironment.

Thus, in the present study, we used machine learning to identify differences in the intestinal microbiome between cases with and without postoperative recurrence of esophageal cancer and compared these with the differences identified using conventional analyses, to ensure accurate assessment of differences. We believe that this approach should provide an effective method for exploring the potential of intestinal bacteria as prognostic markers for postoperative recurrence of esophageal cancer.

## Materials and methods

### Patients

Between January 2017 and September 2018, thoracoscopic resection was attempted in 51 patients with esophageal cancer by the surgical team at the Department of Esophageal Surgery, Showa University Hospital. We included patients with carcinoma of the thoracic esophagus, without serious cardiac or respiratory disease that would preclude safe surgery under general anesthesia; without metastases to other organs, such as the lung or liver; and with tumor stage lower than T4b. Clinicopathological factors were classified according to the UICC-TNM criteria (7th edition).

Stool samples were collected immediately before surgery from patients who underwent neoadjuvant chemotherapy and surgery for esophageal cancer. The outcome was postoperative recurrence and the minimum observation period was 12 months. Patients were divided into two groups according to their prognosis: those who had cancer recurrence after esophageal cancer surgery and those who did not.

This retrospective study was approved by the Ethics Committee of Showa University School of Medicine (Approval No. 2208). All patients provided written informed consent to participate in this study.

### DNA extraction from feces

Fecal samples were collected from each patient within 3 weeks before surgery, using a stool collection kit containing guanidine (TechnoSuruga Laboratory, Shizuoka, Japan). Fecal samples were stored at -80 °C until analysis. DNA for 16S rRNA sequencing was extracted from the fecal samples using a QIAamp PowerFecal Pro DNA Kit (QIAGEN, Hilden, Germany) according to the manufacturer’s instructions.

### Metagenome analysis

Metagenome analysis was performed on a next-generation sequencer (MySeq: Illumina, San Diego, CA, USA) to analyze the 16S V3 and V4 regions of ribosomal RNA genes. The sequence data in FASTQ format were imported into QIIME2 (version 2021.4, https://docs.qiime2.org/2021.4/), quality-controlled with the qiime dada2 plugin and explored for downstream analysis using Feature Table artifacts. A rooted phylogenetic tree, required for alpha diversity analysis, was generated using the theq2-phylogeny plugin. Reads from each sample were rarefied to a depth of 5,000–10,000 to minimize the effect of sequencing depth on alpha and beta diversity measures. Based on the 16S rRNA sequence data obtained, taxonomic and compositional analyses were performed using the plugins q2-feature-classifier, q2-taxa, and the R package qiime2R (https://github.com/jbisanz/qiime2R). The sequence data for the human gut bacteria gene are available in the DDJB database under accession numbers SAMD00651076.

### Statistical analysis

Statistical analysis for the data presented in Fig. 4 was performed using the Mann–Whitney *U* test in R version 4.0.5 (https://www.r-project.org/), running under RStudio 1.4.1106 (https://download1.rstudio.org/desktop/windows/RStudio-1.4.1106.exe). The Kaplan–Meier method was applied for the analysis of data presented in Fig. 6 using the JMP Pro software (SAS, Tokyo, JAPAN).

### Machine learning analysis

Machine learning analysis was performed as previously described [16]. The subjects were divided into recurrence and non-recurrence groups using a random forest model as described before. The intestinal bacterial composition ratio at the genus or species level was used as an explanatory variable, and the presence or absence of recurrence was used as the supervised signal. The scikit-learn library in Python (https://scikit-learn.org/stable/) was used for training. The model was tuned using Optuna (https://optuna.org/) to maximize the area under the receiver operating characteristic (ROC) curve (AUC). Parameter tuning was performed over 1000 iterations. Model accuracy was evaluated using leave-one sample-out cross-validation, which involved 51 iterations, because the dataset included 51 samples. The tuned parameters and fixed random seeds were used in all the iterations. The model was trained using 51 sample combinations, resulting in 51 models.

Feature importance was assessed for all 51 models. Importance scores were used to rank the contribution of the bacterial composition ratios at the genus or species levels. The higher the order of the bacteria, the more important it was deemed for discrimination between recurrence and non-recurrence of esophageal cancer. The number of times each bacterial species appeared in the top 10 in the contribution ranking in the different models was counted, and the species with the highest counts were listed. Owing to the algorithmic randomness inherent in random forests, the impact of this randomness on the contribution ranking based on feature importance was reduced by repeating the above steps four times. Each time, the hyperparameters were set to tuned values, and the random seed was changed.

## Results

### Differences in the abundance of gut microbiome composition at species level between non-recurrence and recurrence group

**Fig. 1:**
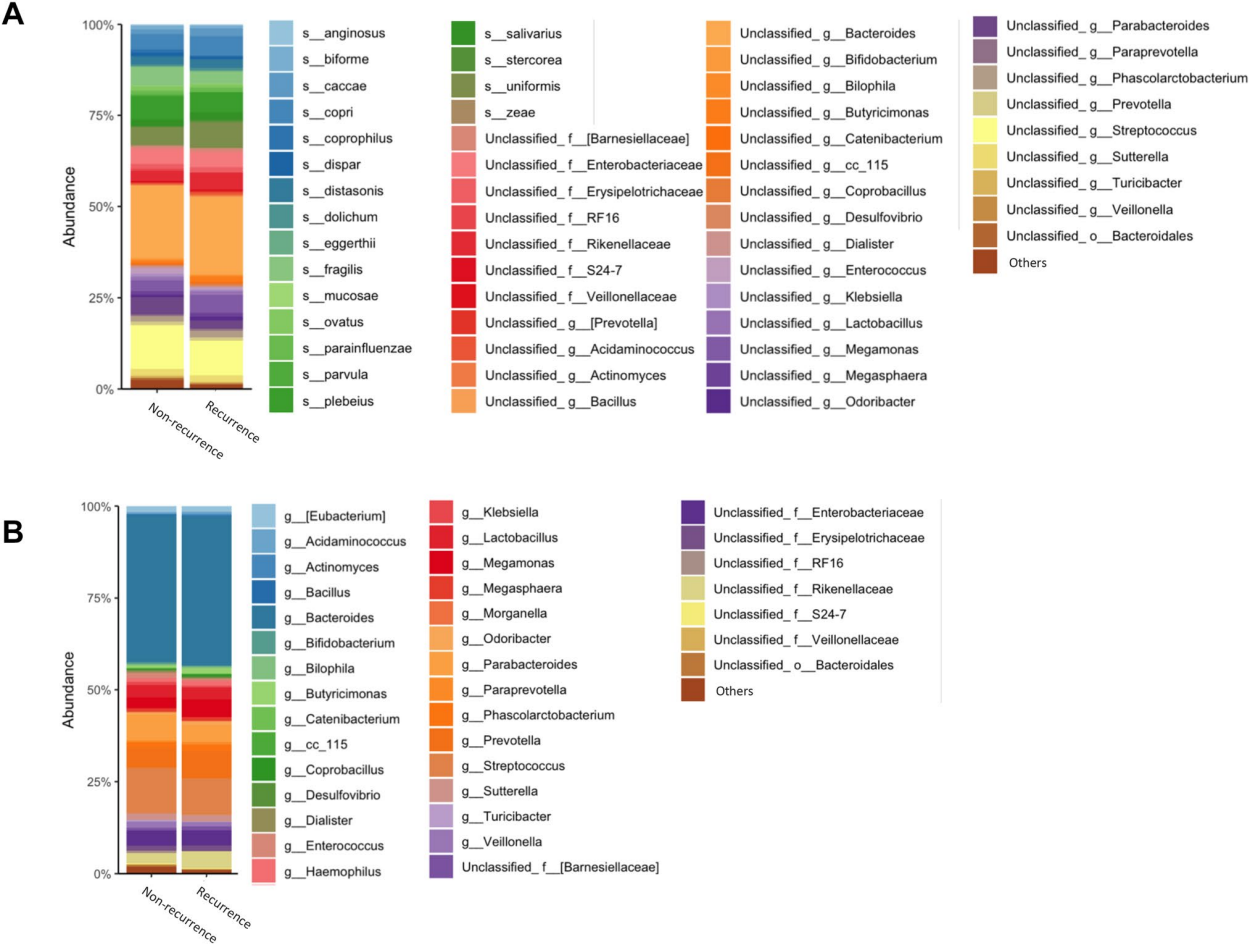
Percentage composition of microbiota profile in patients with esophageal cancer. **A** Relative abundance (%, composition) of bacteria at the species level between the non-recurrence and recurrence groups. Bacteria that were found in more than 0.1% of the cases were summed to 100%. **B** Relative abundance (%, composition) of bacteria at the genus level between the non-recurrence and recurrence groups. Bacteria that were found in more than 0.1% of the cases were summed to 100%

**Fig. 2:**
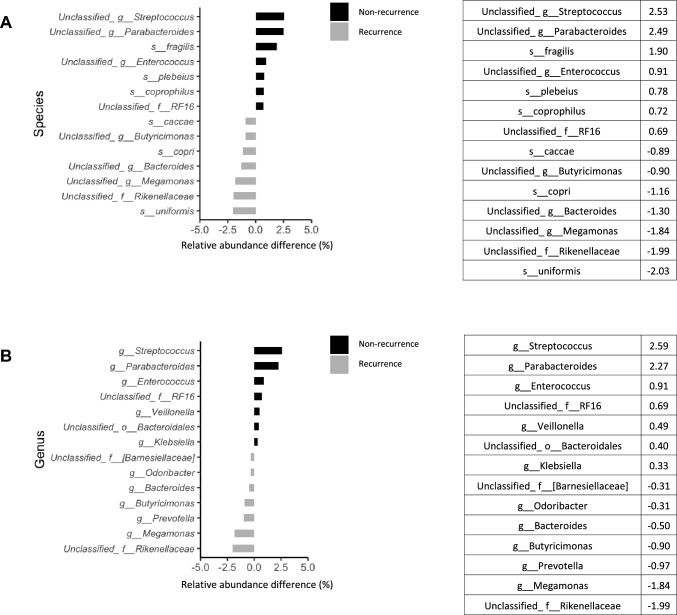
Differences in gut microbiome composition between non-recurrence and recurrence group. We compared the relative abundance difference in bacteria between non-recurrence and recurrence group. **A** Top 7 bacteria by treatment effect at the species level. **B** Top 7 bacteria by treatment effect at the genus level

**Fig. 3:**
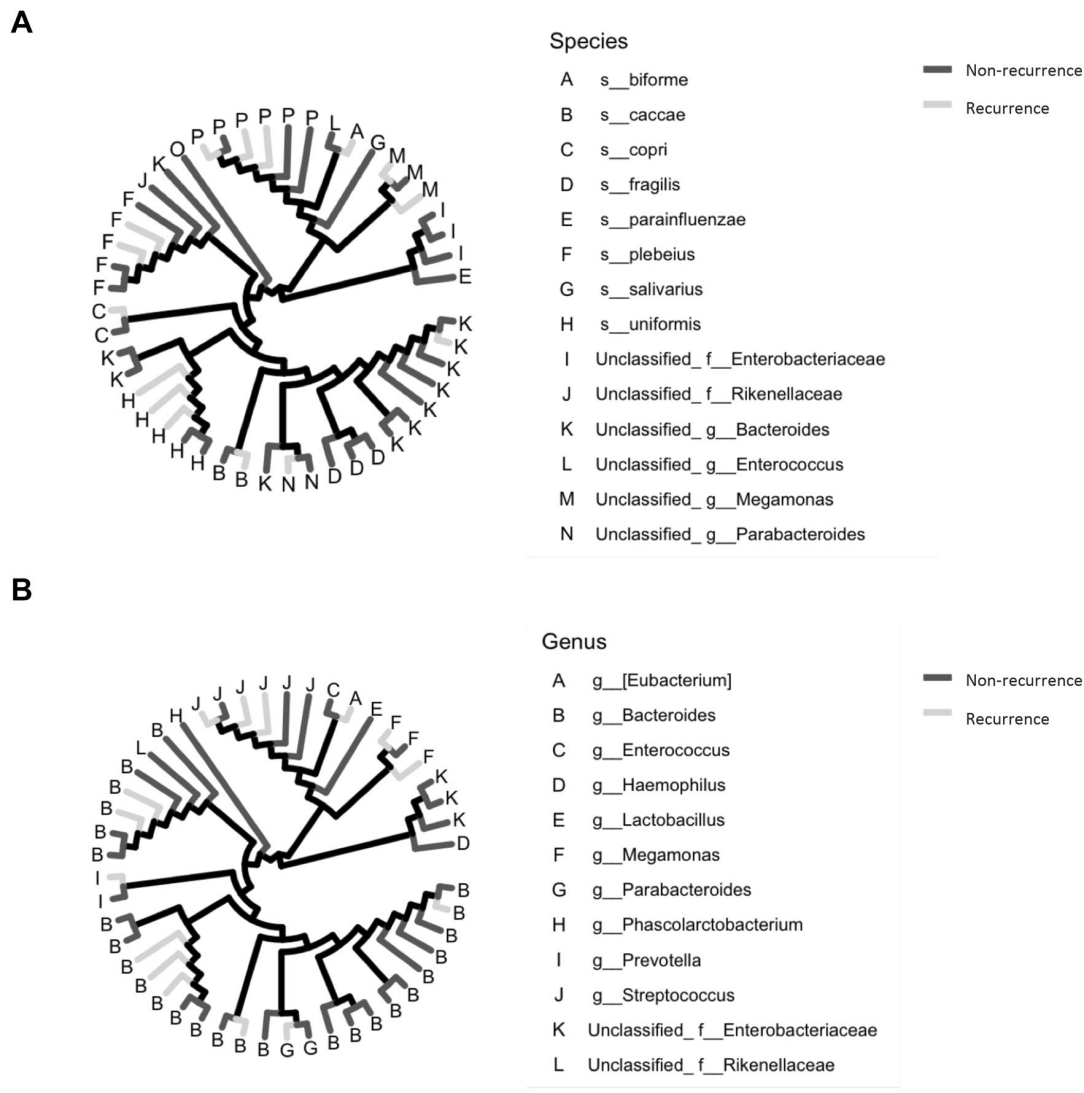
Bacterial tree diagram between non-recurrence and recurrence group. **A** Bacterial tree diagram, with the dark gray and light gray lines indicating the bacteria at the species level between non-recurrence and recurrence group, respectively. **B** Bacterial tree diagram, with the dark gray and light gray lines indicating the bacteria at the genus level between non-recurrence and recurrence group, respectively

We analyzed and compared the intestinal microbiome profile between the group with and the group without postoperative recurrence (Fig. 1A, B). Among the bacteria harvested before surgery from the patients, *Bacteroides uniformis*, *Prevotella copri*, and *Bacteroides caccae* were detected more frequently in patients with postoperative recurrence of esophageal cancer than in those without recurrence (Fig. 2A). *Bacteroides fragilis*, *Bacteroides plebeius*, and *Bacteroides coprophilus* were detected more frequently in patients who did not experience recurrence after esophageal cancer surgery than in those who did (Fig. 2A). In Fig. 3A, the bacteria shown in Fig. 1A are shown in a phylogenetic diagram, with phylogeny color-coded according to the prognosis (non-recurrence vs. recurrence).

### Differences in the abundance of gut microbiome composition at genus level between the non-recurrence and recurrence groups

At the genus level, unclassified Rikenellaceae at family level, *Megamonas*, *Prevotella*, *Butyricimonas*, *Bacteroides*, and *Odoribacter* were detected more frequently in patients with postoperative recurrence of esophageal cancer than in those without recurrence (Fig. 2B). *Streptococcus*, *Parabacteroides*, *Enterococcus*, *Veillonella*, and *Klebsiella* were detected more frequently in patients who did not experience recurrence after esophageal cancer surgery than in those who did (Fig. 2B). In Fig. 3B, the bacteria shown in Fig. 1B are shown in a phylogenetic diagram, with phylogeny color-coded according to the prognosis (non-recurrence vs. recurrence).

### Statistically significant differences in the gut microbiome between the non-recurrence and recurrence groups

**Fig. 4:**
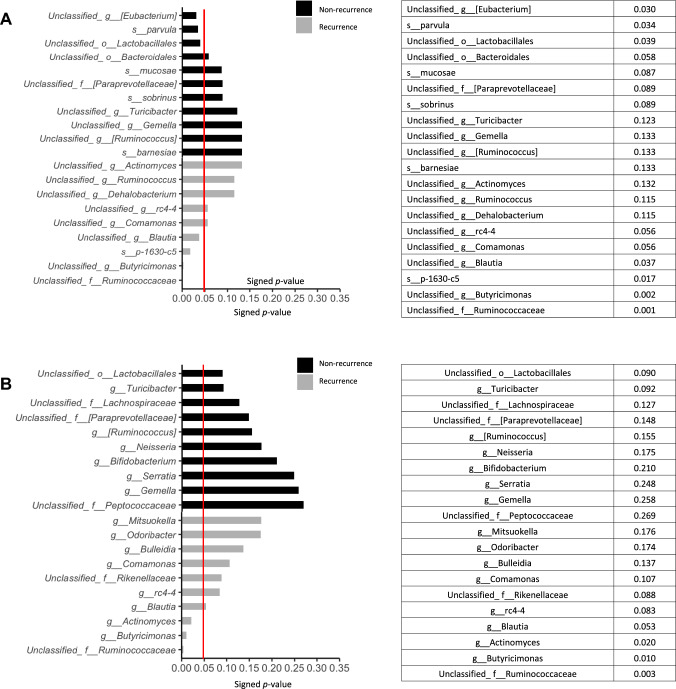
Statistically significant differences in intestinal bacteria. We compared the statistical significance of differences in bacteria between non-recurrence and recurrence group, using the Mann–Whitney U test. The red line indicates a *p*-value of 0.05. **A** Top 10 bacteria at the species level. **B** Top 10 bacteria at genus level

Comparison of the bacterial flora of the groups with and without postoperative recurrence revealed that *Veillonella parvula* was significantly more prevalent in patients who did not experience recurrence after esophageal cancer surgery than in those who did (*p* = 0.034) (Fig. 4A). At the genus level, *Butyricimonas* and *Actinomyces* as were detected significantly more often, at the second and third highest level, respectively, in patients who experienced recurrence after esophageal cancer surgery than in those who did not (*p* = 0.010 and *p* = 0.020, respectively) (Fig. 4B).

### Random forest analysis with hyperparameter tuning

**Fig. 5:**
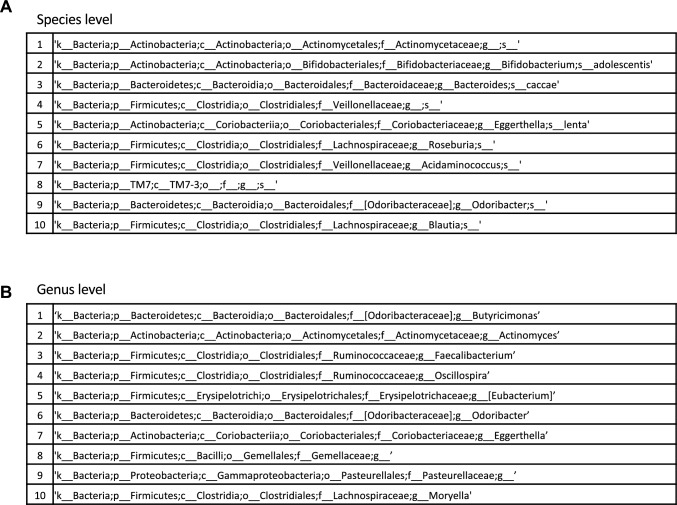
Top 10 gut microbiomes from random forest analysis with hyperparameter tuning. **A** Top 10 bacteria at the species level. **B** Top 10 bacteria at genus level

After calculating the correct ratio, sensitivity, and specificity to identify the group with cancer recurrence using a random forest model, we found that the sensitivity of the model was too low. We recalculated this utilizing 1,000 hyperparameter tunings, which resulted in a sensitivity of more than 50% (Supplementary Fig. 1A, B). The top 10 gut microbiomes from this result are shown at the species and genus levels in Fig. 5A, B. The top-ranking genus in the gut microbiome of the cancer recurrence group according to the machine learning analysis was *Butyricimonas*, which ranked second in the conventional statistical analysis.

### Relative abundance of *Butyricimonas* is associated with differences in survival rate

**Fig. 6:**
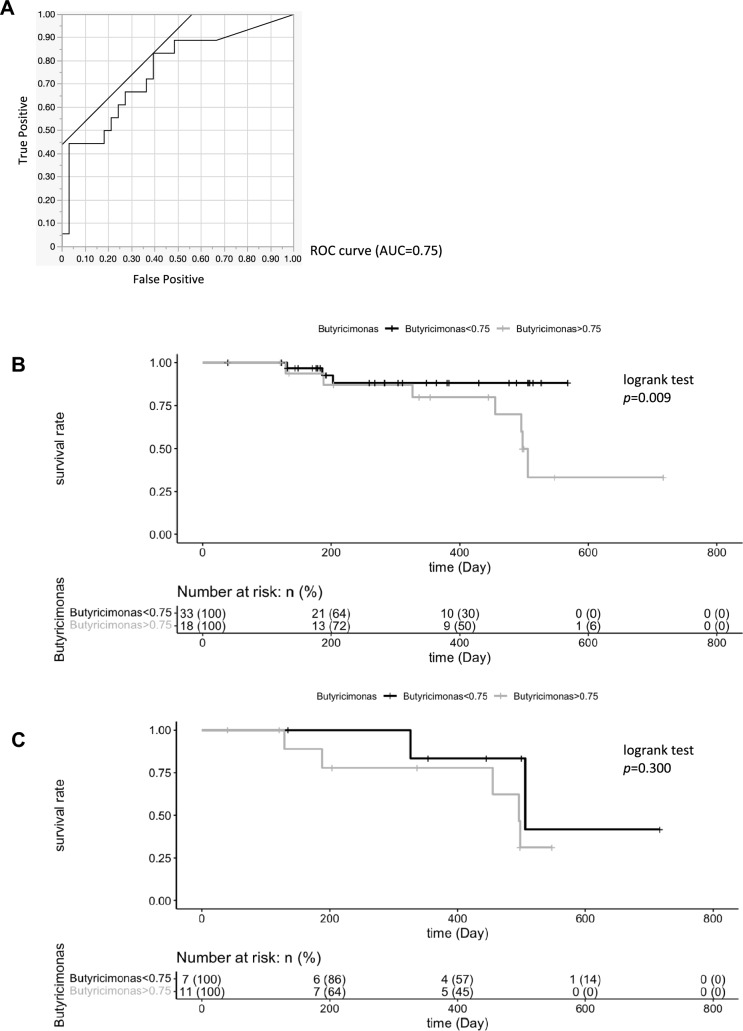
Relative abundance of *Butyricimonas* is associated with differences in survival rate. **A** ROC according to patient with esophageal cancer calculated by cross-validated random forest models. **B** Kaplan–Meier estimates for survival probability based on the abundance levels of *Butyricimonas* in patient with esophageal cancer after surgery. **C** Kaplan–Meier estimates for survival probability based on the abundance levels of *Butyricimonas* in the postoperative recurrence group

The ROC curve for the abundance of *Butyricimonas* among the gut bacteria in patients, overall in terms of patient survival after surgery, yielded an AUC of 0.75 (Fig. 6A). We, thus, compared the survival rates of patients according to the presence or absence of *Butyricimonas*, at an abundance of 0.75% of the total intestinal bacteria, using Kaplan–Meier survival curves. All patients with an abundance of *Butyricimonas* exceeding 0.75% had a significantly higher mortality rate than did those with an abundance of less than 0.75% (*p* = 0.009) (Fig. 6B). Comparison of the survival rates in the group with postoperative recurrence according to the 0.75% abundance of *Butyricimonas* showed no significant difference (*p* = 0.300) (Fig. 6C).

## Discussion

Based on the association between intestinal bacteria and cancer observed in various studies, we hypothesized that some of the differentially present bacteria might be predictive of postoperative prognosis in patients with esophageal cancer. The gut microbiome was examined in patients with and without postoperative recurrence of esophageal cancer at 12 months. The top-10 bacteria with statistically significant differences were compared with the top-10 bacteria identified using hyperparameter-tuned machine learning. Comparison of the intestinal microbiome revealed several species of bacteria in each group that differed from those in the other group. However, only two bacteria—*Butyricimonas* and *Actinomyces*—overlapped between the two approaches at the genus level. Although the reason for the differences in the organisms identified remains unclear, it may be due to the specificity of learning in nonlinear analysis, which may increase the accuracy. Nevertheless, we considered that the bacteria identified using both these approaches would have the best potential for use as accurate biomarkers.

*Bacteroides uniformis* demonstrated the highest abundance at species level in patients with postoperative recurrence of esophageal cancer compared with that in patients without such recurrence. At the species level, *Bacteroides fragilis* was detected more frequently in patients who did not develop postoperative recurrence of esophageal cancer than in those who did. *Bacteroides fragilis* has been reported to be present at significantly higher levels in patients with colorectal cancer than in healthy controls [17]. *Fusobacterium nucleatum*, an oral bacterium, is associated with esophageal cancer [18]. It is known to migrate into esophageal tissues, inducing inflammation and the release of chemokines, such as CCL20, and has been associated with the development and prognosis of cancer recurrence. In our study, *Fusobacterium* was not found to be associated with cancer recurrence, perhaps because it was present in very small numbers and was not detected in most patients.

*Butyricimonas* and *Actinomyces* spp. were detected significantly more frequently in patients with postoperative recurrence of esophageal cancer than in those without recurrence. In the machine learning analysis, only *Butyricimonas* showed a significantly higher abundance, and was the highest ranked differentially present bacterial genus between patients with and those without postoperative recurrence of esophageal cancer.

Furthermore, in this study, *Streptococcus*, *Parabacteroides*, *Enterococcus*, *Veillonella*, and *Klebsiella* were detected more frequently in patients who did not experience recurrence after esophageal cancer surgery than in those who did. *Parabacteroides* has been suggested to have anticancer effects in mouse models of colorectal cancer through suppression of TLR4 and AKT signaling [19]. Therefore, *Parabacteroides* may exert anticancer activity in esophageal cancer via the suppression of TLR4 and AKT signaling. *Streptococcus* is strongly associated with colorectal cancer growth and has been reported to be specifically linked to early rectal cancer progression [20]. In addition, in a mouse colon cancer model, collagenase-degrading bacteria in the genus *Enterococcus*, such as *Enterococcus faecalis*, were reported to be associated with an increased frequency of postoperative tumor development [21]. The differences between these reports and our findings may be due to the different sites of cancer and differences between humans and mouse models.

In our study, *Megamonas*, *Prevotella*, *Butyricimonas*, *Bacteroides*, and *Odoribacter* were detected more frequently in patients with postoperative recurrence of esophageal cancer than in those without the recurrence. *Prevotella* spp. have been reported to be more abundant in patients with colorectal cancer than in healthy individuals [22].

*Butyricimonas* is a butyrate-producing bacterium in the intestinal tract that is less abundant in patients with breast cancer than in healthy controls [23, 24]. *Butyricimonas* was considered a bad bacterium for cancer therapy, associated with a bad response to cancer treatment; however, the results of the present study contradict this notion. Of these genera, only *Butyricimonas* was detected statistically more frequently in patients with postoperative recurrence of esophageal cancer than in those without the recurrence. Interestingly, in the machine learning analysis, *Butyricimonas* was the most sensitive and specific organism for distinguishing between the recurrence and no-recurrence groups. It was also the fourth most common bacterium in terms of amount of bacterium, with the highest difference in bacterial abundance observed between the groups with and without recurrence. Therefore, we hypothesized that *Butyricimonas* spp. may be strongly associated with the postoperative recurrence of esophageal cancer. In the intestine, *Butyricimonas* mainly produces short-chain fatty acids, such as butyric acid. Butyric acid has been reported to decrease tumor cell growth [25]. Butyric acid inhibits histone deacetylation. The resultant increased acetylation leads to downregulation of the calcineurin–NFATc3 pathway, which is involved in tumor cell proliferation. In contrast, butyric acid has immunosuppressive effects and has been reported to increase tolerance to CTLA-4 blockade and the percentage of regulatory T cells (Tregs) in the cancer immune system [26]. In our study, *Butyricimonas* was detected more frequently in patients with postoperative cancer recurrence, possibly because butyric acid produced by *Butyricimonas* suppresses cancer immunity. As evident from the survival curves, patients with an abundance of *Butyricimonas* greater than 0.75% had a significantly higher mortality rate than those with an abundance of less than 0.75% of this organism. However, in the group with postoperative recurrence, survival did not differ between patients with an abundance of *Butyricimonas* greater than or less than 0.75%. Therefore, *Butyricimonas* may be involved in postoperative patient survival.

*Actinomyces* was also detected significantly more often in patients with postoperative recurrence of esophageal cancer than in those without the recurrence. *Actinomyces* is a bacterium that mainly colonizes the oral cavity [27]. However, *Actinomyces* spp. did not differ between the postoperative recurrence and non-recurrence groups in terms of bacterial abundance.

In conclusion, by comparing the gut microbiome immediately before esophageal cancer surgery, we found that *Butyricimonas* was more prevalent in patients with esophageal cancer recurrence by 12 months postoperatively than in those without the recurrence. This difference was significant in both the statistical and machine learning analysis. This indicates that one organism in the intestinal microbiome can be used as a biomarker for predicting postoperative esophageal cancer recurrence. In future, a prospective evaluation of whether this intestinal bacterium can be used to predict postoperative recurrence is warranted.

### Supplementary Information

Below is the link to the electronic supplementary material.

Supplementary file1 (PDF 371 kb)

## Data Availability

The data that support the findings of this study are available from the corresponding author, upon reasonable request.
