# Supplementary material for: Butyricimonas is a key gut microbiome component for predicting postoperative recurrence of esophageal cancer
Source: Cancer Immunol Immunother. 2024 Jan 27;73(2):23. doi: 10.1007/s00262-023-03608-y (PMC10821974; doi:10.1007/s00262-023-03608-y)
Supplement: Supplementary file 1 — Supplementary file1 (PDF 371 kb) [file 262_2023_3608_MOESM1_ESM.pdf]

Supplementary Fig. 1

A

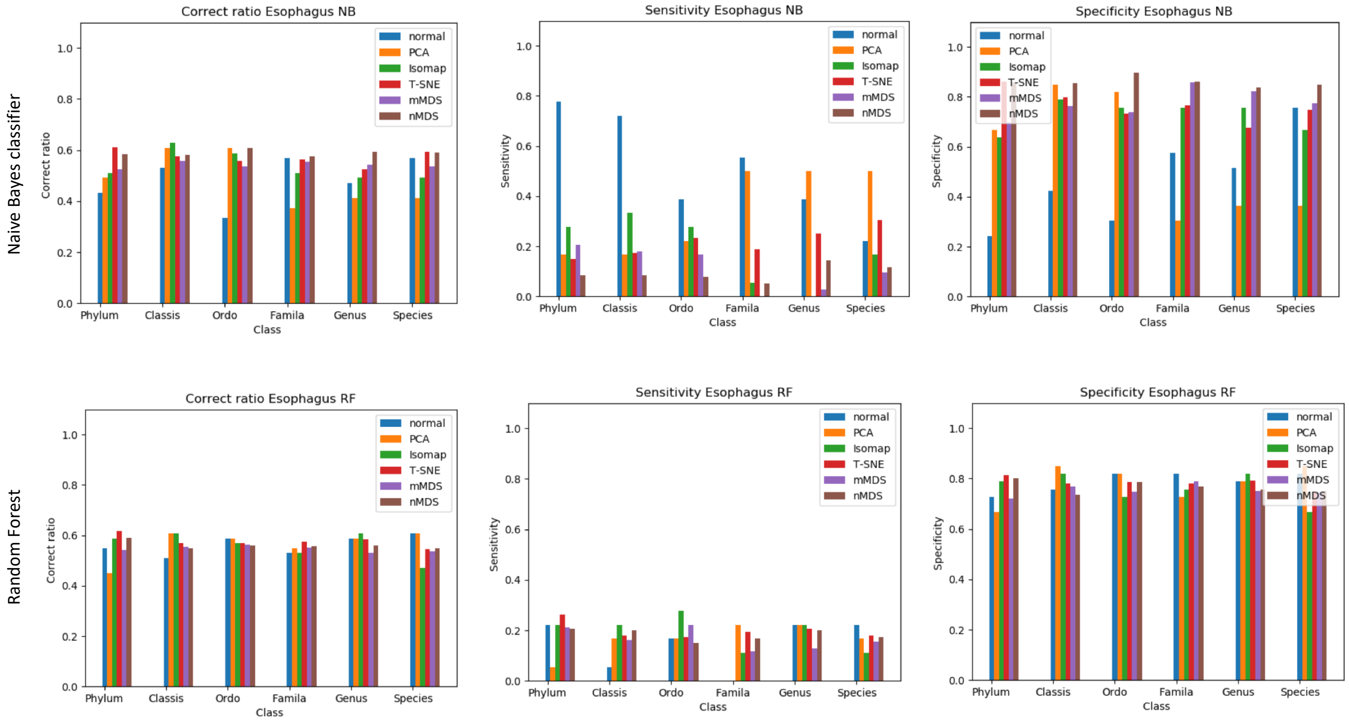

B

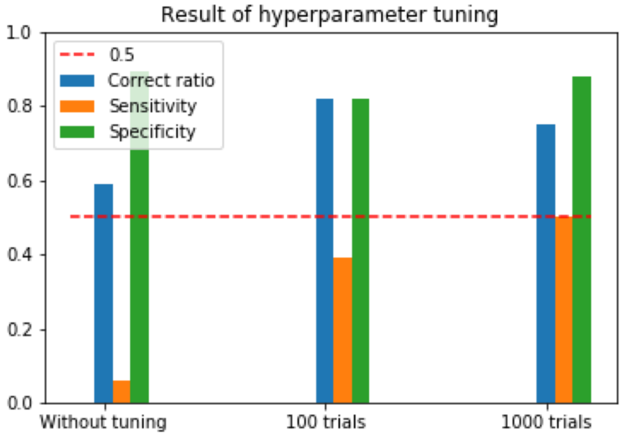

Recalculating the correct ratio, sensitivity, and specificity to identify the group with cancer recurrence.
(A) Calculating the correct ratio, sensitivity, and specificity to identify the group with cancer recurrence using a naïve bayes classifier (upper) or a random forest model (lower). (B) Recalculating the correct ratio, sensitivity, and specificity to identify the group with cancer recurrence utilizing hyperparameter tunings.
